# Associations Between Lifestyle Factors, Oral Health Behaviors, and Glycemic Control in Type 2 Diabetic Patients

**DOI:** 10.3390/jcm14020450

**Published:** 2025-01-12

**Authors:** Vanessa Bolchis, Iulia Alexa, Nicoleta A. Toderas, Ramona Dumitrescu, Ruxandra Sava-Rosianu, Octavia Balean, Vlad Tiberiu Alexa, Simona Popescu, Daniela Jumanca, Atena Galuscan, Iosif Ilia, Doina Chioran

**Affiliations:** 1 Translational and Experimental Clinical Research Centre in Oral Health, Department of Preventive, Community Dentistry and Oral Health, University of Medicine and Pharmacy “Victor Babes”, 300040 Timisoara, Romania; vanessa.bolchis@umft.ro (V.B.); iulia.alexa@umft.ro (I.A.); dumitrescu.ramona@umft.ro (R.D.); balean.octavia@umft.ro (O.B.); vlad.alexa@umft.ro (V.T.A.); jumanca.daniela@umft.ro (D.J.); galuscan.atena@umft.ro (A.G.); 2 Clinic of Preventive, Community Dentistry and Oral Health, Department I, University of Medicine and 13 Pharmacy “Victor Babes”, Eftimie Murgu Sq. no 2, 300041 Timisoara, Romania; 3 Department of Dentistry, Faculty of Dental Medicine, “Vasile Goldis” Western University of Arad, 310045 Arad, Romania; 4 Department of Psychology, Faculty of Sociology and Psychology, West University of Timisoara, 300223 Timisoara, Romania; nicoleta.toderas01@e-uvt.ro; 5 Second Department of Internal Medicine, “Victor Babes” University of Medicine and Pharmacy, 300041 Timisoara, Romania; popescu.simona@umft.ro; 6 Department of Diabetes, “Pius Brinzeu” Emergency Hospital, 300723 Timisoara, Romania; 7 Faculty of Physical Education and Sport, “Aurel Vlaicu” University of Arad, 310130 Arad, Romania; iosif.ilia@uav.ro; 8 Faculty of Dental Medicine, “Victor Babeș” University of Medicine and Pharmacy from Timisoara, 9 Revolutiei 1989 Ave., 300070 Timisoara, Romania; chioran.doina@umft.ro

**Keywords:** T2DM, oral health behaviors, lifestyle factors, glycemic control, HbA1c, prevention

## Abstract

**Introduction:** T2DM mellitus (T2DM) is a major global health issue associated with significant morbidity, mortality, and economic burden. While the role of lifestyle factors in glycemic control is well-established, the influence of oral health behaviors remains underexplored. **Objective:** This study aimed to investigate the interplay between lifestyle habits, oral health behaviors, and glycemic control in patients with T2DM. **Methodology:** A cross-sectional study was conducted on 132 patients (66 men and 66 women) with T2DM at the Pius Brînzeu Emergency Hospital in Timișoara, Romania. Data on smoking, physical activity, alcohol consumption, tooth brushing frequency, and dental visits were collected using structured questionnaires, and glycemic control was assessed through HbA1c measurements. Statistical analyses, including Pearson correlations and linear regression, were performed. **Results:** Among men, HbA1c levels were negatively associated with exercise frequency (ß = −0.26, *p* < 0.05) and education level (correlation coefficient −0.27, *p* < 0.05), and positively associated with dental visits and tooth brushing frequency (correlation coefficient 0.26, *p* < 0.05). In the combined analysis, education level positively correlated with both dental visits (correlation coefficient 0.24, *p* < 0.01) and alcohol consumption (correlation coefficient 0.22, *p* < 0.05). **Conclusions:** These findings underscore the importance of integrating oral health and lifestyle interventions into diabetes management to optimize patient outcomes.

## 1. Introduction

Type 2 diabetes mellitus (T2DM) is a chronic metabolic disorder characterized by persistent hyperglycemia due to relative or absolute insulin deficiency, affecting the metabolism of carbohydrates, proteins, and fats. In addition to its systemic complications, T2DM significantly impacts oral health, manifesting through conditions such as periodontal diseases, tooth loss, delayed wound healing, candidiasis, dry mouth, and burning mouth syndrome, which may serve as early indicators for undiagnosed diabetes or markers for glycemic control in diagnosed patients [1]. Acording to the WHO the expected values for normal fasting blood glucose concentration are between 70 mg/dL (3.9 mmol/L) and 100 mg/dL (5.6 mmol/L). The American Diabetes Association (ADA) defines normal fasting plasma glucose as below 100 mg/dL (5.6 mmol/L), with values between 100 and 125 mg/dL (5.6 to 6.9 mmol/L) indicating impaired fasting glucose (pre-diabetes). Diagnosis of T2DM is based on specific glucose thresholds, including fasting plasma glucose (FPG) levels of ≥126 mg/dL (7.0 mmol/L), 2-h plasma glucose (2-h PG) levels of ≥200 mg/dL (11.1 mmol/L) during a 75-g oral glucose tolerance test (OGTT), or an HbA1c level of ≥6.5% (48 mmol/mol). In individuals presenting with classic hyperglycemic symptoms or during a hyperglycemic crisis, a random plasma glucose level of ≥200 mg/dL (11.1 mmol/L) confirms the diagnosis. These thresholds are crucial for understanding glycemic control and provide essential context for analyzing diabetes-related data [2,3]. As a progressive condition, it negatively affects health-related quality of life, leads to severe comorbidities, and increases the risk of premature mortality. In addition to its health impact, this disease places a significant economic burden on individuals, healthcare systems, and society. However, research shows that it can be effectively prevented through lifestyle changes, underscoring the importance of early prevention efforts. Moreover, the positive effects of lifestyle interventions can last for several years. Despite more than two decades of evidence supporting the effectiveness of these approaches, healthcare systems still struggle to implement scalable, individual-level support for lifestyle changes in routine practice [4,5,6,7].

Optimal health behaviors include avoiding smoking, limiting alcohol intake and engaging in regular physical activity. These habits, along with balanced nutrition and managing mental stress, are linked to stronger immune function, particularly through enhanced natural killer cell activity, the body’s first line of defense [8,9].

For individuals with diabetes, lifestyle factors play an even more critical role. Men with diabetes who adopt healthier behaviors and experience fewer microvascular complications are often those with higher educational levels. Among diabetic women, those with more education tend to perceive themselves as healthier, regardless of their actual medical status. However, diabetic patients with poor metabolic control and lower educational attainment frequently report more complications, mental health issues, sick leave, and reduced physical activity, highlighting the importance of both education and lifestyle in managing the condition. Additionally, social class and gender disparities further complicate outcomes, with lower-class diabetic women facing a higher mortality risk compared to both non-diabetic women and diabetic men from similar backgrounds [9,10].

In addition to its systemic effects, T2DM significantly impacts oral health, with a well-established bidirectional relationship between diabetes and periodontal disease. Poor glycemic control exacerbates periodontal inflammation through mechanisms such as oxidative stress, delayed wound healing, and heightened inflammatory responses. Conversely, untreated periodontitis amplifies systemic inflammation, complicating glycemic management and increasing the risk of diabetes-related complications. Decompensated diabetic patients often exhibit a diminished response to non-surgical periodontal therapy, such as scaling and root planing, due to delayed tissue healing and altered immune function. This underscores the critical role of achieving glycemic control to enhance periodontal treatment outcomes. Lifestyle choices, including proper oral hygiene, balanced nutrition, and regular physical activity, are essential for addressing the interconnected impacts of T2DM and periodontitis. Adopting healthier habits not only aids in controlling blood glucose levels but also mitigates systemic and oral health challenges associated with diabetes [11,12,13,14].

Adopting healthier habits, such as improving diet and increasing physical activity, is essential for controlling blood glucose levels and reducing the global prevalence of diabetes. A healthy lifestyle, as defined by the World Health Organization, involves daily habits and behaviors that reduce the risk of disease or premature death by addressing critical factors such as balanced nutrition and regular exercise. This multifaceted concept encompasses various aspects of life, including diet, physical activity, sleep, stress management, and substance use, all of which are influenced by social, economic, and environmental factors. By adopting these preventive measures, individuals can mitigate chronic diseases, enhance overall well-being, and improve long-term health outcomes. In the case of periodontitis, immune responses are key to the severity of inflammation, as bacteria trigger the release of inflammatory mediators like TNF-α and interleukin-1, which contribute to tissue and bone damage [15,16].

Poor blood sugar control in diabetes also induces oxidative stress, slowing tissue repair in the gums and making infections harder to heal, which worsens periodontal conditions. Additionally, complications like reduced blood flow further impede recovery in diabetic patients, increasing susceptibility to periodontal damage. These connections highlight the significant impact of lifestyle on both oral and systemic health [9,15,17,18].

Good oral hygiene habits include brushing teeth at least twice daily, visiting the dentist annually, using interdental devices like floss, and limiting snacking and cariogenic foods. These habits are associated with healthy food consumption, regular exercise, and vitamin use, particularly among adolescents. Physical activity supports better brushing habits; while smoking and alcohol consumption are linked to poorer oral health. Poor oral hygiene leads to plaque buildup and worsens periodontal health, while frequent brushing is linked to reduced sugar intake. Although diabetic patients tend to have poor oral health behaviors, the absence of a control group limits conclusions about the connection between oral health and type-2 diabetes. Interestingly, even non-diabetic periodontitis patients have been found to have elevated fasting blood glucose levels [19,20].

A complementary literature review analyzed 52 studies using Web of Science, with keyword co-occurrence analysis in VOSviewer identifying emerging links between lifestyle factors, oral health, and glycemic control.

This study aimed to investigate the interplay between T2DM, oral health behaviors, and lifestyle factors such as smoking, alcohol consumption, and physical activity. By exploring how these elements intersect with diabetes management, we sought to identify key areas for improving the quality of life and comprehensive care of diabetic individuals. The absence of similar studies in Romania underscores the importance of this research in addressing local knowledge gaps and providing a foundation for developing targeted public health strategies tailored to the Romanian context.

## 2. Background of the Study

The current research aimed to explore the relationships between T2DM, oral health behaviors, and lifestyle factors starting from a detailed analysis of existing literature. The process of screening the literature was initiated by using Web of Science, a globally recognized academic database that provides access to thousands of high-quality research articles across multiple disciplines, including medicine, public health, and life sciences. Through the Web of Science Core Collection, which includes highly cited journals, conference proceedings, and patent records a basic search was conducted focusing on three main keywords: T2DM, Oral Health Behaviors, and Lifestyle Factors.

After the initial screening of 52 relevant scientific articles that focused on the core themes of this research were selected. These articles were chosen based on their relevance, citation impact, and the quality of the journals in which they were published. Web of Science’s advanced filtering options, including citation network analysis and the ability to track emerging trends in the field, helped ensure that the selected studies provided a comprehensive overview of the latest research.

To better understand the relationships between key concepts in the literature, VOSviewer (version 1.6.20), a powerful visualization software designed to map co-occurrences of keywords from these selected articles was utilized. VOSviewer allows for the identification and analysis of relationships between key terms, providing a clear perspective on emerging trends and significant areas of interest in T2DM research.

The co-occurrence analysis of keywords helped to identify the most critical themes within the selected literature. Through this mapping, strong connections between terms such as T2DM, obesity, physical activity, insulin resistance, oral health behaviors, and smoking were discovered. These connections highlight the complex interplay between lifestyle factors and the management of diabetes. This approach not only emphasized the individual importance of each factor but also showed how they interact to influence glycemic control and the prevention of diabetes-related complications.

Thus, by analyzing the co-occurrence and visualizing the relationships between these concepts, the importance of this subject in the context of public health and clinical management could be underscored. The findings provide important insights into how lifestyle, smoking, and oral health behaviors directly influence the management of T2DM.

**Figure 1:**
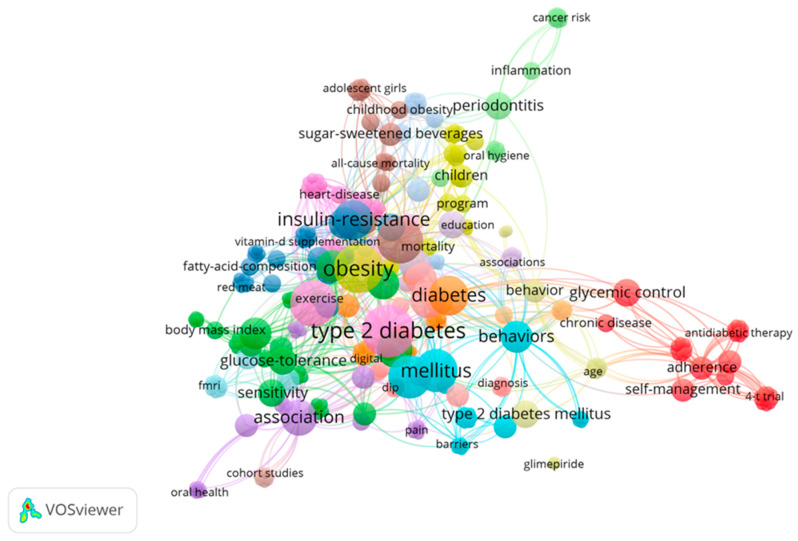
Clustered Network Visualization of Key Concepts in T2DM Research.

The co-occurrence map generated from 52 Web of Science articles highlights key thematic connections in T2DM research (Figure 1). Central terms like T2DM and obesity emphasize their critical relationship, with strong links to insulin resistance and glycemic control, underscoring their importance in disease management. The map also reveals the significance of lifestyle factors, such as physical activity and diet, which play a crucial role in improving health outcomes. Peripheral but notable themes include oral health behaviors and inflammation, illustrating emerging research areas exploring how chronic conditions like periodontitis impact diabetes. This comprehensive visualization reflects the need for an integrative approach to diabetes care, incorporating both lifestyle and systemic health factors.

**Figure 2:**
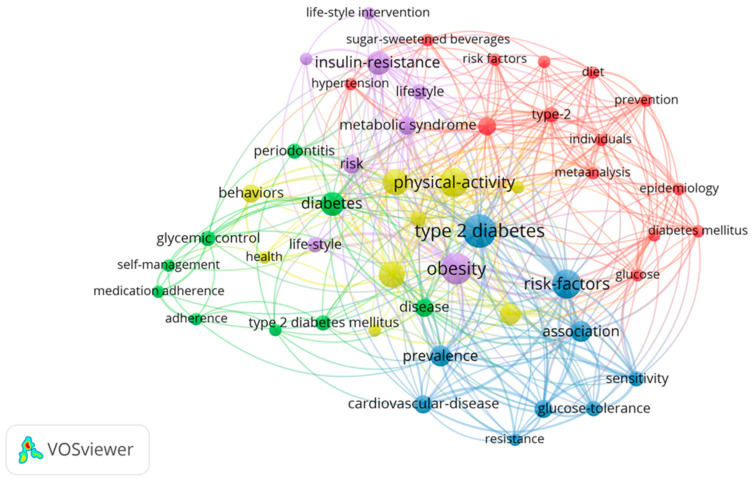
Refined Co-occurrence Map Highlighting Central Themes in T2DM Research.

In the next phase, the co-occurrence map was refined by raising the threshold to include only keywords with a minimum of 3 occurrences, narrowing the analysis to 51 key terms (Figure 2). This adjustment helped to highlight the most central and frequently discussed themes in the literature. Core topics such as diabetes management, lifestyle interventions, and risk factors remained prominent, reflecting the ongoing research focus. The streamlined analysis also reinforced the importance of behavioral and physiological aspects of T2DM care, emphasizing how lifestyle changes and patient engagement play critical roles in disease management.

**Figure 3:**
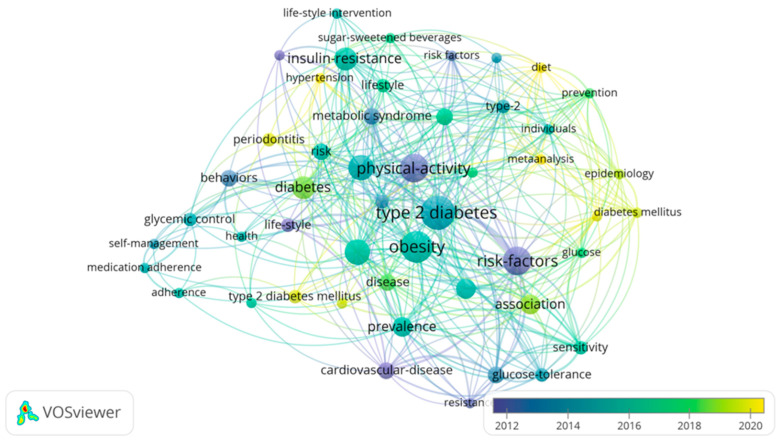
Temporal Evolution of Research Focus in T2DM.

The final analysis focused on identifying trends in the evolution of research topics over time. Using a time-overlay visualization in VOSviewer, the color gradient in the map—from blue to yellow—reveals the shifts in research emphasis from 2012 to 2020 (Figure 3). Early research (highlighted in blue and green) predominantly focused on general risk factors for T2DM, such as obesity, insulin resistance, and cardiovascular disease. These foundational topics laid the groundwork for understanding the broader scope of diabetes-related complications and the role of lifestyle in disease progression.

As the years progressed, newer research (depicted in yellow) shifted towards more specific interventions and public health strategies, with increasing attention on epidemiology, meta-analyses, and preventive measures like diet and lifestyle interventions. This shift underscores a growing recognition of the importance of early prevention and tailored interventions in diabetes care. Additionally, keywords related to self-management, medication adherence, and periodontitis show the expanding focus on patient-centered care and the integration of oral health into diabetes management.

This temporal analysis not only highlights the dynamic nature of diabetes research but also demonstrates a forward-looking trend toward prevention and holistic care. By tracing these shifts in research focus, we gain a deeper understanding of how the field has evolved, paving the way for more comprehensive and effective approaches to managing T2DM.

## 3. Materials and Methods

### 3.1. Study Design

This cross-sectional, observational and correlational study was conducted at the Outpatient Diabetes Care Facility of the Pius Brînzeu County Emergency Hospital in Timișoara, Romania, between February and April 2024. A total of 132 patients with T2DM (66 women and 66 men) participated, reflecting a diverse demographic representative of diabetic patients in the region. All participants provided informed consent prior to their inclusion in the study, ensuring ethical compliance and voluntary participation. None of the participants were involved in the design or development of the research, maintaining the integrity and objectivity of the study process. The research was conducted in full alignment with the ethical principles outlined in the 2013 revision of the Declaration of Helsinki, which governs ethical standards for research involving human subjects. Furthermore, the study received formal approval from the Ethics Committee of the University of Medicine and Pharmacy Victor Babes, Timisoara, Romania, under protocol number 05/30.01.2024, affirming its adherence to rigorous ethical guidelines. For drafting the manuscript, GenAI tools have been used solely for English editing, given the fact that none of the authors are native English speakers.

This study was conducted in Romania, focusing on the western region, which reflects the country’s notable regional and local disparities in human development. The research examines areas with varying levels of socioeconomic development, as indicated by education attainment, income levels, and access to healthcare services. The western region, characterized by a mix of industrial, agricultural, and service-based economic activities, includes urban centers like Timișoara that serve as regional economic hubs with relatively high education levels and well-developed healthcare infrastructure, particularly in Timiș County, which specializes in chronic care such as diabetes management. In contrast, rural areas in the region face challenges related to poverty, limited connectivity, and reduced access to healthcare and resources. Geographically, the combination of plains and mountainous terrains influences lifestyle behaviors, including agricultural practices and recreational activities, providing a nuanced context for studying the interplay between lifestyle, oral health behaviors, and glycemic control in diabetic patients [21,22].

The participants were drawn from the western region of Romania, where the prevalence of diabetes is estimated at 8.23%, as reported by the PREDATORR study. To ensure that the study captured a broad spectrum of experiences, a stratified random sampling method was employed, balancing representation based on age, gender, and residential environment (urban vs. rural). This approach minimized selection bias and enhanced the applicability of the findings to the wider population of diabetic patients.

### 3.2. Inclusion/Exclusion Criteria

The study included individuals aged 18 and older, all diagnosed with T2DM. Exclusion criteria encompassed patients with type 1 diabetes, those who did not provide informed consent, individuals with severe cognitive or psychiatric impairments that hindered their ability to consent, pregnant or breastfeeding women, and those who had undergone major surgery or experienced significant physical trauma within the previous six months.

### 3.3. Data Collection

The data collection process was conducted by a team of three healthcare professionals, including a diabetologist (S.P.) and two dentists (V.B. and R.D). Participants were provided with an information sheet detailing the study’s objectives and procedures, addressing any questions they had. Participation was voluntary, and individuals who met the inclusion criteria and consented to participate signed a written consent form. Subsequently, they were asked to complete a self-administered questionnaire while waiting for their medical appointment. Completing the questionnaire required approximately 8 to 15 min. The questionnaire was developed following a thorough review of existing literature and prior studies [23,24]. It consisted of sections assessing lifestyle factors, oral hygiene behaviors, and demographic and clinical characteristics. The lifestyle factors section included questions on smoking habits, alcohol consumption, and the frequency of physical activity. Oral hygiene behaviors were evaluated by examining the frequency of toothbrushing, the use of fluoride toothpaste, and the utilization of auxiliary cleaning methods, such as dental floss or interdental brushes. The questionnaire used was developed based on the WHO adult questionnaire, ensuring its validity and relevance for assessing health behaviors and lifestyle in the target population [25]. Clinical data, such as glycated hemoglobin (HbA1c) levels, were extracted from participants’ medical records to ensure accuracy and standardization. Demographic data collected included age, gender, place of residence (urban or rural), diabetes duration, and education level. This structured and comprehensive approach ensured the collection of high-quality data on the relationships between lifestyle factors, oral health behaviors, and glycemic control among individuals with T2DM, allowing for a nuanced analysis of these interrelated variables.

### 3.4. Variables and Study Outcomes

The primary dependent variable in this study was glycemic control, measured by glycated hemoglobin (HbA1c) levels obtained from participants’ medical records. Secondary dependent variables included oral health behaviors, specifically the frequency of tooth brushing and dental visits. Independent variables encompassed lifestyle factors such as smoking status, alcohol consumption, and frequency of physical activity; demographic characteristics, including age, gender, place of residence (urban or rural), and educational level; as well as clinical characteristics, such as the duration of diabetes and the use of oral hygiene aids like dental floss or interdental brushes. This framework allowed for a comprehensive analysis of the relationships between lifestyle, oral health behaviors, and glycemic control among diabetic patients.

### 3.5. Statistical Analysis

The statistical analysis combined insights from a comprehensive literature review with the evaluation of empirical data. The primary objective of the statistical tests was to comprehensively analyze and elucidate the intricate relationships between various lifestyle factors, oral health behaviors, and HbA1c levels, while simultaneously investigating potential gender-specific differences within the study population. These analyses aimed to provide a deeper understanding of how these variables interact and influence one another, contributing to the overall health outcomes of individuals with diabetes.

Descriptive statistics summarized the demographic and behavioral characteristics of the 132 participants, while Pearson correlations assessed relationships between variables such as exercise frequency, education level, dental visits, and HbA1c levels. A split-file analysis by gender further highlighted specific trends, including significant negative correlations between HbA1c and exercise frequency or education among men, and a positive correlation between education and dental visits among women. Linear regression models identified predictors of HbA1c variability, with exercise frequency emerging as the strongest negative predictor. All analyses were conducted using SPSS version 23, with statistical significance set at *p* < 0.05.

## 4. Results

To further ensure representativeness, the sample reflected the regional demographic profile of diabetic patients, with approximately equal numbers of participants from urban (53%) and rural (47%) areas, mirroring the distribution reported in national health surveys. Additionally, age distribution ranged from 34 to 87 years, with the majority of patients in the 50–65 age group, a demographic most affected by T2DM. This stratification provided a nuanced understanding of the diverse challenges faced by diabetic individuals across different socioeconomic and geographical backgrounds, strengthening the external validity of the study’s findings (Table 1).

The values of Skewness and Kurtosis for the variables of interest fall within the parametric range (−2, 2), indicating that the dataset follows a normal distribution and is parametric. The participants were equally distributed by gender, with 66 males and 66 females (N = 132). The majority of participants had completed either secondary education (10 classes) or high school (12 classes), accounting for 64.4% (N = 85) of the sample, followed by 20.5% (N = 27) who had a maximum of 8 years of education (gymnasium).

The mean age of the sample was 63.45 years (SD = 10.48), with the youngest participant aged 34 and the oldest aged 87. Regarding alcohol consumption, 50% of participants (N = 66) reported occasional or monthly consumption, while 9.1% (N = 12) consumed alcohol daily, and 33.3% (N = 44) abstained entirely.

In terms of exercise frequency, defined as any type of physical activity performed for an extended duration, the majority of participants (49.2%, N = 65) reported being inactive. Among the remaining participants, 22.7% (N = 30) engaged in physical activity 1–2 times per week, 15.9% (N = 21) exercised 3–4 times per week, and 12% (N = 16) reported exercising 5 or more times per week.

Oral health was assessed through two variables: the frequency of dental visits and the frequency of tooth brushing. Most participants (47.7%, N = 63) reported not visiting the dentist in the past year, while 44.7% (N = 59) visited once or twice, and a minority of 7.6% (N = 10) visited three or more times annually. Regarding tooth brushing habits, 38.6% (N = 51) brushed their teeth twice daily, 31.1% (N = 41) brushed once daily, 16.7% (N = 22) brushed less than once daily, and 13.6% (N = 18) brushed more than twice daily (Table 2).

### 4.1. Gender-Specific Correlations

For gender specific correlation the Pearson correlation was used after splitting the groups between females and males. The results show that regarding males, there’s a significative positive correlation between tooth brushing frequency and dental visits R (66) = 0.26, *p* < 0.05, with a small effect size of r^2^ = 0.06, meaning the frequency of tooth brushing increases as males attend more dental check-ups. Regarding the value of HbA1, there are two variable that correlate negatively to this. First, there is a negative significative correlation between frequency of exercise and value of HbA1c, R (66) = −0.26, *p* < 0.05, with a small effect size of r^2^ = 0.06, meaning that the value of HbA1c increases as the frequency exercise per week decreases. Secondly, there is a negative significative correlation between HbA1c and education level R (66) = −0.27, *p* < 0.05 with a small effect size of r^2^ = 0.06, meaning that the HbA1c value increases as the education level o participants gets smaller (Table 3).

For the female subgroup (N = 66), the analysis identified a single significant positive correlation. Specifically, the frequency of dental visits was positively correlated with the level of education achieved R (66) = 0.25, *p* < 0.05 with a small effect of r^2^= 0.06, meaning that women with higher education also have higher rate of dental visits in the last year.

Although no other statistically significant correlations were identified within this subgroup, this result underscores the potential influence of education on oral health behaviors. Women with higher educational attainment may have increased awareness of the importance of regular dental care, access to better resources, or a stronger inclination toward preventive health behaviors, contributing to this observed trend.

When analyzing at the correlation between the variables without taking into account the gender, we found the following results regarding studies: there is a positive significative correlation between level of studies and frequency of dental visits R (132) = 0.24, *p* < 0.01, with a small effect size of r^2^= 0.05, meaning that overall, when the level of studies increases so does the frequency of dental visits. Additionally, the last significative result we found was regarding level of studies and alcohol intake, we found a positive significative correlation of R (132) = 0.22, *p* < 0.05 with a small effect of r^2^ = 0.04. These findings highlight the potential influence of education on health-related behaviors, such as oral hygiene and lifestyle habits. While the effect sizes are small, the consistent association between higher education levels and both dental visits and alcohol intake suggests the need for further research to explore the underlying mechanisms and potential socioeconomic or cultural factors influencing these behaviors.

To explore the nuances between variances and variables, a split-file analysis based on gender was conducted. A linear regression analysis was performed with HbA1c as the dependent variable and the following predictors: frequency of dental visits, exercise frequency, alcohol consumption, and education level, analyzed separately for men and women.

The relationships between these predictors and HbA1c levels are further illustrated in the linear regression model displayed in Table 3, which provides a visual representation of their relative contributions to the variability in glycemic control.

For the whole sample the results of the linear regression analysis shows that the level of studies, frequency of dental visits and exercise frequency significantly predict the variability of HbA1c F (3, 120) = 3.23, *p* < 0.05. These variables predict in proportion of 0.075% of the total variability of HbA1c, R^2^ = 0.07. The strongest negative predictor was level of exercise with a β = −0.17, *p* = 0.05 explaining 2.89% of the total variability of HbA1c, R^2^ = 0.02 (Table 4).

The results of the linear regression analysis for men showed us that there are some significant predictors of HbA1c, F (5, 50) = 2.49, *p* < 0.05, with the strongest negative significant predictor for men being dental visit frequency, β = −0.26, *p* = 0.05, explaining 5% of the total variability of HbA1c for men, R^2^ = 0.05.

No significant results were observed for women in relation to any of the chosen predictors (dental visits, level of exercise, level of education, teeth brushing frequency, and alcohol consumption).

### 4.2. Correlations Between Age and Oral Health Status, Glycemic Control (HbA1c) and Lifestyle Factors

To further explore the nuances in the relationships between the variables, a Pearson correlation analysis was conducted based on age. The only significant correlations identified within this study sample were between age and level of education, as well as age and frequency of dental visits. The results showed a significative correlation R (106) = −0.33, *p* < 0.01, with a medium to large effect size R^2^ = 0.11, between age distribution and the level of studies, meaning as the age increases, the level of studies decreases. Another significative correlation was found between age and frequency of dental visits, with a R (112) = −0.32, *p* < 0.01, with a medium to large effect size of R^2^ = 0.10, meaning as the age of participants increased, the frequency of dental visits decreased. As indicated in Table 5, no other significant correlations were observed between age and the remaining variables.

## 5. Discussion

This study explored the intricate relationships between T2DM mellitus, lifestyle factors, and oral health behaviors, providing new insights into the determinants of glycemic control (HbA1c) and their variability among diabetic patients in Romania. Our findings underscore the critical role of lifestyle and education in managing T2DM mellitus, aligning with global research trends while addressing gaps specific to the Romanian population.

The primary goal of diabetes management is achieving optimal glycemic control, yet our study revealed that a substantial proportion of patients with T2DM had poor glycemic control, with a mean HbA1c of 7.95% (SD = ±1.52). This finding aligns with earlier studies that reported high rates of poor glycemic control, ranging from 65% to 81.9% [26,27], further emphasizing the persistent challenge in managing diabetes effectively. However, the proportion remains significantly higher compared to developed countries, such as the United States, where only 12.9% of patients have poor glycemic control [28]. This disparity likely reflects differences in healthcare infrastructure, patient education, and access to resources. For instance, in developed settings, uniform clinical guidelines, comprehensive health insurance, and robust primary care systems likely contribute to better glycemic outcomes. In contrast, our findings suggest that gaps in awareness, adherence to lifestyle modifications, and limited access to dental and medical care may hinder effective management in our study population. This is further supported by the observed negative correlation between HbA1c levels and education level, as well as exercise frequency, highlighting the pivotal role of socioeconomic and behavioral factors in glycemic control.

Previous research has highlighted the relationship between glycemic control and lifestyle factors [29,30].

Similarly, our study identified significant associations between glycemic control and key lifestyle components such as exercise and medical adherence. These findings underscore the critical importance of patient awareness and adherence to disease management and lifestyle modifications in achieving effective diabetes control.

Our study sheds light on the role of physical activity in glycemic control, contributing valuable data to the existing body of research. Previous studies have consistently linked physical inactivity to higher glucose levels and poor glycemic control [31,32,33].

Our findings confirm this association, demonstrating a significant negative correlation between exercise frequency and HbA1c levels, particularly among male participants (R (66) = −0.26, *p* < 0.05). This suggests that consistent physical activity is associated with better glycemic outcomes. However, a concerning 49.2% of our participants (N = 65) were categorized as physically inactive, with only 22.7% (N = 30) engaging in exercise 1–2 times weekly and 12% (N = 16) exercising five or more times weekly. Furthermore, adherence to both dietary and exercise regimens was limited, with just 30 participants adhering to recommended practices, highlighting a significant gap in lifestyle management. These results emphasize the urgent need for targeted interventions to promote regular exercise and dietary adherence among patients with T2DM. Barriers to physical activity, including lack of awareness, accessibility, or motivation, must be addressed through structured educational programs and individualized support systems. The observed associations reinforce the broader evidence advocating for lifestyle modifications as critical components of effective diabetes management. Interventions tailored to the unique challenges faced by this population could significantly enhance self-management capabilities, reduce HbA1c levels, and ultimately improve health outcomes.

The observed correlations between higher education levels and improved health behaviors, such as more frequent dental visits, reflect the importance of education in fostering awareness and access to preventive care. These results are consistent with existing literature, which highlights education as a significant determinant of health outcomes. This study reinforces the influence of sociodemographic factors on glycemic control, in line with previous findings that younger and less-educated individuals tend to exhibit higher HbA1c levels compared to their older, better-educated counterparts [34]. In our analysis, a significant negative correlation was observed between education level and HbA1c values, indicating that higher education is associated with better glycemic control. This disparity underscores the importance of educational interventions targeted at enhancing diabetes self-management, particularly among younger and less-educated populations, to mitigate the observed gaps in glycemic outcomes.

For men, the negative correlation between education and HbA1c levels emphasizes the broader impact of educational attainment on metabolic control, suggesting that educational interventions might be particularly beneficial for improving diabetes management among this subgroup. Physical activity emerged as a strong negative predictor of HbA1c levels, reinforcing the importance of exercise in diabetes care. This finding aligns with global evidence that regular physical activity improves insulin sensitivity and glycemic control. However, nearly half of the participants reported being inactive, highlighting a critical area for targeted public health interventions. Gender-specific strategies may be necessary, as exercise frequency was significantly associated with HbA1c in men but not in women. This difference suggests potential sociocultural or biological factors influencing health behaviors, which warrant further investigation.

The present study revealed significant gaps in oral hygiene behaviors and dental care among patients with diabetes, paralleling findings from another research. Specifically, 38.6% of our participants brushed their teeth twice daily, slightly below the 49.3% average reported in a systematic review of diabetes patients’ oral hygiene practices [35]. Moreover, only 7.6% of participants in our study visited a dentist three or more times annually, with 47.7% not visiting a dentist at all in the past year. This aligns with broader findings showing just over half (54%) of diabetes patients worldwide visit a dentist annually [36], a rate substantially lower than the general population in high-income countries such as England (73%), the U.S. (64%), and Australia [35].

Regarding oral health behaviors, our diabetic group showed suboptimal practices compared to international standards but slightly better adherence to dental visits compared to other Romanian study [37]. For instance, while Sadeghi et al. [38] reported that 83% of participants attended regular dental checkups, our study found that 65.27% of diabetic participants did so, a significant improvement compared to previous local data but still indicative of barriers to consistent preventive care. Additionally, brushing frequency in our diabetic cohort was less frequent than ideal, with only a minority adhering to the recommended twice-daily brushing regimen.

Our findings further support the notion that barriers to regular dental care—such as lack of perceived need, limited awareness, and accessibility issues—are prevalent among diabetic patients. This is particularly concerning given the established bidirectional relationship between periodontal health and glycemic control, where poor oral health can exacerbate diabetes complications. The low adherence to flossing practices, with only a quarter of global diabetes patients flossing daily [35], mirrors our results, suggesting a critical need for improved education on the importance of interdental cleaning.

Interestingly, the frequency of dental visits also predicted HbA1c variability in men, highlighting the link between oral health and systemic health. Periodontal inflammation, which is exacerbated by poor glycemic control, can worsen diabetes outcomes, creating a bidirectional relationship. The present findings suggest that men who visit the dentist more frequently may experience better metabolic control, possibly due to reduced inflammation or heightened health awareness. This connection underscores the need for integrating oral health education and care into diabetes management programs.

For women, while no significant predictors of HbA1c were identified, the positive correlation between education and dental visits suggests a critical role for educational attainment in shaping preventive health behaviors. Women with higher education levels may have better access to dental care and a greater understanding of its importance in overall health. This finding aligns with existing research suggesting that women’s health behaviors are influenced by socioeconomic and educational factors, further emphasizing the need for targeted educational programs.

Overall, this study highlights the multifaceted nature of diabetes care, emphasizing the interplay between lifestyle factors, education, and oral health. These findings suggest that effective management of T2DM mellitus requires a holistic approach that integrates lifestyle interventions, educational support, and oral health care. Given the limited focus on these factors in Romania, our results provide a strong foundation for developing tailored public health strategies and patient-centered care models. Future research should aim to explore the underlying mechanisms driving these relationships and evaluate the effectiveness of integrative interventions in improving outcomes for diabetic patients.

This study has several limitations that should be considered when interpreting the findings. First, the cross-sectional design limits the ability to infer causal relationships between lifestyle factors, oral health behaviors, and glycemic control, highlighting the need for longitudinal studies to confirm these associations. Second, reliance on self-reported data for variables such as exercise, alcohol consumption, and oral hygiene behaviors introduces potential reporting bias, as participants may have under- or overreported their habits. The study’s focus on a single outpatient facility in western Romania may also restrict the generalizability of findings to other regions or populations with differing socioeconomic or healthcare contexts. Additionally, the relatively small sample size, though reflective of the local population, may have reduced the statistical power to detect smaller effects, especially in subgroup analyses. The *p*-value requirement for inclusion (*p* < 0.05) was emphasized; however, it should be noted that this predictor is at the upper threshold of significance with a *p*-value of *p* = 0.05. Consequently, the conclusion should be interpreted with caution.

Another notable limitation is the absence of clinical oral examination data, such as DMFT, plaque index, or gingival index, which could have provided deeper insights into participants’ oral health and its relationship with lifestyle and glycemic control. While self-reported oral health behaviors were valuable, they lack the objectivity and detail clinical measures could offer. This omission may have limited the study’s ability to explore specific mechanisms linking oral and systemic health. Future research should address these limitations by incorporating larger, more diverse samples, longitudinal designs, and both self-reported and objective measures of lifestyle and health behaviors for a more comprehensive understanding.

## 6. Conclusions

This study highlights the intricate relationships between lifestyle factors, oral health behaviors, and glycemic control in patients with T2DM mellitus (T2DM). The findings emphasize that both lifestyle and oral health practices play crucial roles in managing glycemic levels, with education emerging as a consistent determinant influencing both metabolic outcomes and preventive health behaviors.

These results underscore the necessity of integrative approaches in diabetes care, combining lifestyle modifications with oral health interventions to optimize metabolic control and improve overall health outcomes in the present sample. Future research should further explore the underlying mechanisms driving these associations and assess the efficacy of combined educational and behavioral interventions. Tailored strategies addressing socioeconomic disparities and fostering patient engagement are essential to achieving comprehensive care for individuals with T2DM.

## Figures and Tables

**Table 1:** Descriptive Statistics for Key Variables in the Study Sample (N = 132).

Variables	N (%)
Gender	
Male	66 (50.0)
Female	66 (50.0)
Age	
34–44	4 (2.7)
45–54	18 (16.2)
55–64	33 (29.8)
65–74	45 (40.5)
75–87	12 (10.8)
Missing age	20 (15.2)
Residence	
Rural area	51 (38.6)
Urban area	81 (61.4)

**Table 2:** Descriptive Statistics for Behavioral, Demographic, and Health Variables (N = 132).

Variables	Mean	Std. Deviation
Studies	1.88	0.55
HbA1C	7.95	1.51
Alcohol consumption	1.73	0.62
Dental visit	1.59	0.62
Exercise	2.87	1.24
Age	63.45	10.48
Tooth brushing	2.49	0.92
Gender	1.50	0.50

**Table 3:** Correlation Matrix of Lifestyle Factors, Oral Health Behaviors, and HbA1c Levels Among Participants (N = 66).

	Men			Women	Overall	
	Tooth Brushing Freq.	Exercise	Studies	Dental Visits	Alcohol Consumption	Dental Visits
Dental visit	0.26 *	0.02	0.15	-	0.10	-
Exercise	0.06	-	0.07	0.05	−0.10	0.04
Alcohol consumption	0.00	−0.04	0.24	0.08	-	0.10
Studies	0.15	0.07	-	0.25 *	0.22 *	0.24 **
HbA1C	−0.07	−0.26 *	−0.27 *	−0.09	−0.13	−0.16

*p* * < 0.05; *p* ** < 0.01.

**Table 4:** Linear Regression Analysis Predicting HbA1c Levels Based on Education, Dental Visits, and Exercise Frequency.

	HbA1c		
	Beta	Std Err.	*p*
**Overall**			
Studies	−0.09	0.24	0.28
Dental visits	−0.15	0.22	0.08
Exercise	−0.17 *	0.12	0.05
Alcohol Consumption	−0.79	0.23	0.40
Tooth Washing	−0.05	0.15	0.56
**Men**			
Alcohol consumption	0.12	0.28	0.34
Studies	−0.24	0.32	0.07
Dental visits	−0.26 *	0.32	0.05
Tooth washing	0.07	0.21	0.56
Exercise	−0.21	0.18	0.09
**Women**			
Alchool consumption	−0.20	0.40	0.11
Dental visits	−0.12	0.33	0.35
Tooth Washing	−0.18	0.23	0.32
Exercise	−0.12	0.19	0.32

Note: * *p* < 0.005.

**Table 5:** Correlations between age and socio-demographic and clinical parameters.

	Age	HbA1c	Studies	Alchool Consumption	Exercise Freqeuncy	Dental Visit	Teeth Washing
Pearson Correlation	1.00	0.03	−0.33	−0.12	−0.10	−0.33	0.17
Sig. (2-tailed)		0.78	0.00	0.22	0.30	0.00	0.06
N	112.00	111.00	106.00	102.00	112.00	112.00	112.00

## Data Availability

The data presented in this study are available on request from the corresponding author.
